# Progress of RAGE Molecular Imaging in Alzheimer’s Disease

**DOI:** 10.3389/fnagi.2020.00227

**Published:** 2020-08-04

**Authors:** Yanyan Kong, Cuiping Liu, Yinping Zhou, Jingxuan Qi, Chencheng Zhang, Bomin Sun, Jiao Wang, Yihui Guan

**Affiliations:** ^1^PET Center, Huashan Hospital, Fudan University, Shanghai, China; ^2^Laboratory of Molecular Neural Biology, School of Life Sciences, Shanghai University, Shanghai, China; ^3^Department of Neurosurgery, Center for Functional Neurosurgery, Ruijin Hospital, Shanghai Jiao Tong University School of Medicine, Shanghai, China

**Keywords:** AD, RAGE, PET, [^18^F]-FPS-ZM1, senile plaques, neurofibrillary tangles

## Abstract

Alzheimer’s disease (AD) is a progressive neurodegenerative disease characterized by senile plaques (SPs), which are caused by amyloid beta (Aβ) deposition and neurofibrillary tangles (NFTs) of abnormal hyperphosphorylated tau protein. The receptor for advanced glycation end products (RAGE) binds to advanced glycation end products deposited during vascular dysfunction. Alzheimer’s disease may occur when RAGE binds to Aβ and releases reactive oxygen species, further exacerbating Aβ deposition and eventually leading to SPs and NFTs. As it is involved in early AD, RAGE may be considered as a more potent biomarker than Aβ. Positron emission tomography provides valuable information regarding the underlying pathological processes of AD many years before the appearance of clinical symptoms. Thus, to further reveal the role of RAGE in AD pathology and for early diagnosis of AD, a tracer that targets RAGE is needed. In this review, we first describe the early diagnosis of AD and then summarize the interaction between RAGE and Aβ and Tau that is required to induce AD pathology, and finally focus on RAGE-targeting probes, highlighting the potential of RAGE to be used as an effective target. The development of RAGE probes is expected to aid in AD diagnosis and treatment.

## Introduction

Alzheimer’s disease (AD) is the first major neurodegenerative disease with irreversible, occult, and rapid progression. With aging of the population, AD has become a major disease affecting public health (Nebel et al., 2018; Bo et al., 2019). The etiology and pathogenesis of AD are not fully understood, and currently, there is no specific treatment. More importantly, early diagnosis of AD is limited. The cost of treatment and care for AD is enormous, imposing a heavy burden on patients, families, and the society. Therefore, brain function imaging, developed on the basis of brain metabolism research targeting AD pathogenesis, plays an increasingly important role in the study of pathological processes in the AD brain.

The pathological features of AD are senile plaques (SPs), containing neurotoxic amyloid beta (Aβ) as the main component, and neurofibrillary tangles (NFTs), with abnormally activated tau as the main component in nerve cells. Neurofibrillary tangles and SPs are currently recognized as the earliest pathological changes in AD, with SPs reaching their maximum deposition in the early stage of AD, termed the “capping effect,” which allows for amyloid plaque imaging *in vivo*. Tracking the slow progress of AD is difficult (Dubois et al., 2018). Therefore, an in-depth exploration of AD pathogenesis with the development of new radioactive probes that detect pathological changes earlier to Aβ deposition is currently a hotspot in AD research.

The receptor for advanced glycation end products (RAGE) belongs to the immunoglobulin superfamily of cell surface molecules and is situated in the major histocompatibility complex class III locus (Xue et al., 2011; Han et al., 2014). It binds to its ligand, advanced glycation end products (AGEs), through its V-type region, which is a key site that mediates intracellular signal transduction (Kim et al., 2013; Abedini et al., 2018). While mild hypoperfusion can increase the levels of neuronal Aβ and NFTs, expressed as paired helix filaments, increasing evidence shows that RAGE levels are significantly elevated in patients with AD and AD models (Cai et al., 2016; Chellappa and Rani, 2020; Paudel et al., 2020). Receptor for advanced glycation end products-mediated Aβ-injured tight junctions may also be associated with a variety of intracellular signal transduction pathways, Ca^2+^, or inflammatory damage (Nelson et al., 2016; Cai et al., 2017; Sole et al., 2019). Further, immunohistochemical evidence shows that the distribution of RAGE abnormalities is consistent with that of NFTs and SPs. In addition, glycosylated tau can induce significant oxidative stress and cause neuronal insufficiency or death (Srikanth et al., 2011; Cai et al., 2016). Receptor for advanced glycation end products may play an important role in the occurrence and development of AD, yet its underlying mechanism is still unclear. Thus, it is necessary to lay emphasis on the role of RAGE in AD pathology.

### Early Progression of AD

The etiology of AD is complex, and there are currently no specific drugs and methods to treat AD. Many drugs can only achieve remission (Wong et al., 2019). Positron emission tomography (PET), as a molecular imaging technique, can reflect pathological changes at the molecular level and can non-invasively detect the distribution of radionuclides in the body, which reflects physiological, biochemical, metabolic, and receptor changes, as well as gene expression and other abnormal changes (Hannestad, 2018; Mankoff and Katz, 2018). Thus, it is an important auxiliary tool for AD research. At present, there are several types of AD PET imaging agents (Bao et al., 2017) targeting glucose metabolism, receptors, or transmitters, Aβ, Tau protein, neuroinflammation, and monoamine oxidase. However, these agents have certain limitations for the early diagnosis of AD.

In recent years, research on AD has mainly focused on the two major pathological features of AD: Aβ and tau. However, although some individuals show Aβ or Tau deposition as detected on medical images, they exhibit no dementia symptoms (Hardy and Selkoe, 2002). Moreover, studies have shown that Aβ deposition is slow and protracted, likely lasting over 20 years, while the association of Aβ accumulation with cognitive impairments is weak (Villemagne et al., 2013). Additionally, the current probes cannot distinguish among the six subtypes of Tau protein, and their off-target effects are more serious (Robertson et al., 2017). Therefore, finding new targets and developing the corresponding probes for AD are particularly important for AD research.

**FIGURE 1:**
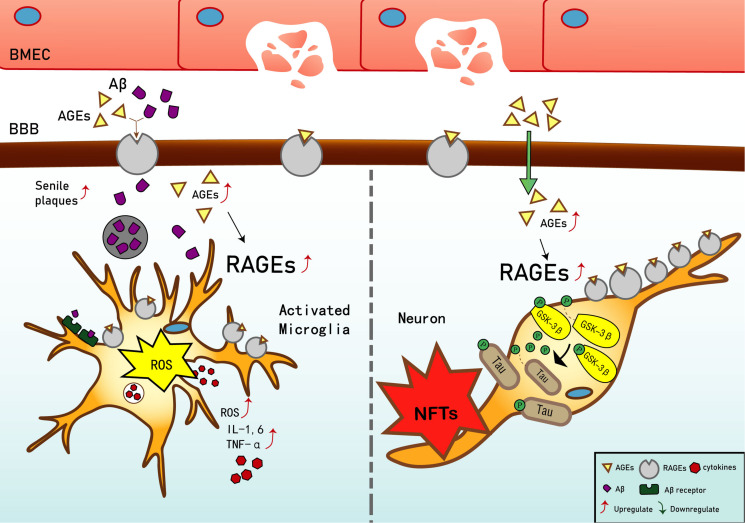
Receptor of advanced glycation end products (RAGE) mediates the possible mechanism of Alzheimer’s disease (AD). Interaction of RAGE with advanced glycation end products (AGEs) and β-amyloid (Aβ) allows them to cross the blood–brain barrier and enter the brain. Aβ deposition in the brain promotes the expression of tumor necrosis factor α (TNF-α), interleukin (IL)-1, and IL-6, and exacerbates the inflammatory response. Increased binding of Aβ to RAGE after entering the brain can also upregulate RAGE expression, resulting in increased release of reactive oxygen species (ROS) and upregulation of Aβ expression, which then promotes the generation of senile plaques (SPs). The upregulation of RAGE exacerbates phosphorylation of tau protein and promotes the generation of neurofibrillary tangles (NFTs). AGEs and RAGE phosphorylate glycogen synthase kinase 3 (GSK-3β), which, in turn, exacerbates the phosphorylation of tau protein and promotes the generation of NFTs. Upregulation of Aβ and tau protein levels promote the occurrence and development of AD.

Current studies have shown that in early vascular dysfunction of AD, inflammatory mediators, such as tumor necrosis factor alpha (TNF-α), in brain microvascular endothelial cells (BMECs), are released, thus increasing cerebral vascular permeability (Qiu et al., 2016), enabling AGEs and other neurotoxicants to cross the blood–brain barrier (BBB) and cause AGE deposition. This leads to a significant upregulation of RAGE in BMECs (Liang et al., 2015), which leads to an inflammatory response by vascular endothelial and nerve cells, activates the release of reactive oxygen species (ROS), which promotes oxidative stress, and results in the secretion of nitric oxide synthase and further increases Aβ deposition in the brain. Aβ increases the activation of microglia, which, in turn, accelerates nerve vessel dysfunction. Neuronal dysfunction promotes the pathogenesis of NFTs, thus causing the formation of additional SPs and NFTs, disturbing the balance in the chemical components of the neuro-microenvironment. This further promotes neuronal dysfunction, injury, and loss (Wells et al., 2015; Cai et al., 2016) (see Figure 1). Based on the above, RAGE and AGEs could play an important role in the early pathological changes of AD.

### AGE-Related RAGE Processes and NFTs

**FIGURE 2:**
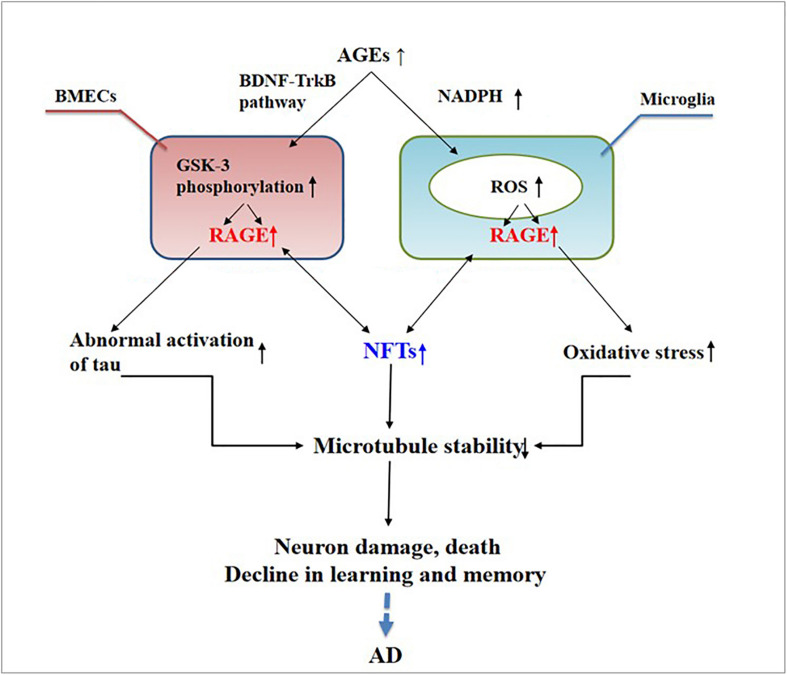
Receptor of advanced glycation end products (RAGE) mediates the possible mechanism of neurofibrillary tangle (NFT) formation in the pathogenesis of dementia complicated with Alzheimer’s disease.

Abnormally activated tau is the main component of NFTs, and NFT deposition in the hippocampus and entorhinal cortex is correlated with the severity of behavioral degeneration in the progression of dementia (Saint-Aubert et al., 2017). Advanced glycation end products are the final products of the non-enzymatic glycation of proteins, which is irreversible. The non-enzymatic saccharification processes accompanying neuronal metabolism have far-reaching effects despite the slow and insignificant cell damage they cause (Kamynina et al., 2018). In AD, AGEs have been shown to induce tau hyperphosphorylation in SK-N-SH cells, primary hippocampal neurons, and rat brains through the RAGE/GSK-3 pathway (Li X.H. et al., 2012; Son et al., 2012). As AGEs downregulate the brain-derived neurotrophic factor–tyrosine receptor kinase B pathway in rat brains and N2A cells (Li X.H. et al., 2012), they could activate glycogen synthase kinase 3 at Ser9, thus regulating its phosphorylation, which was found to be a trigger of tau hyperphosphorylation (Wu et al., 2019). Simultaneously, *in situ* techniques have shown that the major structures recognized by anti-AGE antibodies, hydroxymethyl lysine (CML) and glycosylated precursor hexitol-lysine, increase in the NFTs of patients with AD. In these patients, CML colocalizes with the tau protein. Immunostaining experiments have shown that almost all AGE-immunoreactive neurons contain the hyperphosphorylated tau protein, confirming the role of AGE aggregation in early NFT formation and neuronal degeneration (Qi et al., 2017) (see Figure 2). As an increase in AGEs causes an upregulation of RAGE, the connection between AGEs and NFTs indicates a strong link between RAGE and tau hyperphosphorylation.

The deposition of AGEs in the brain participates in the pathogenesis of AD through RAGE and cross-links with NFTs. This deposition activates microglia and nicotinamide adenine dinucleotide phosphate oxidase, leading to ROS release and the formation of peroxynitrite, a potent oxidant of proteins, lipids, and DNA (Nam et al., 2012), ultimately causing nerve destruction. Therefore, treating AGEs may become a new way to treat AD.

### Role of the Interaction Between RAGE and Aβ in AD

A growing body of evidence suggests that RAGE is an important regulator of Aβ neurotoxicity. Aβ-damaged BMECs and the destruction of the BBB may be new characteristic pathological changes in AD (Lv et al., 2014). In AD, RAGE expression is significantly upregulated in areas where Aβ is deposited (Wang et al., 2009). Receptor for advanced glycation end products is a pattern recognition receptor, and Aβ, as one of its ligands, was shown to interact with it (Paudel et al., 2020); however, the specific mechanism underlying this interaction and its role in patients with AD need further clarification.

The interaction of RAGE with Aβ activates inflammatory signaling pathways, releases ROS to produce oxidative stress, and causes neuroinflammation, thus inducing the dysfunction of mitochondria and neurons (Deane et al., 2008), as well as changes in various signaling mechanisms such as the mitogen-activated protein kinase pathway (Deane, 2012). Further, RAGE accelerates the uptake and transport of Aβ, which causes Aβ to cross the BBB and enter the central nervous system through endocytosis (Deane et al., 2003), causing cerebrovascular dysfunction, eventually leading to neurovascular inflammation and subsequent synaptic toxicity (Deane and Zlokovic, 2007), thereby affecting the normal activity of the central nervous system (Zhang et al., 2011; Galasko et al., 2014; Wang et al., 2014; Cai et al., 2016; Fang et al., 2018). The interaction between RAGE and Aβ is harmful to the body. Studies have found that, in transgenic mice with defective RAGE expression, Aβ in the brain is completely inhibited from crossing the BBB (Deane and Zlokovic, 2007).

**FIGURE 3:**
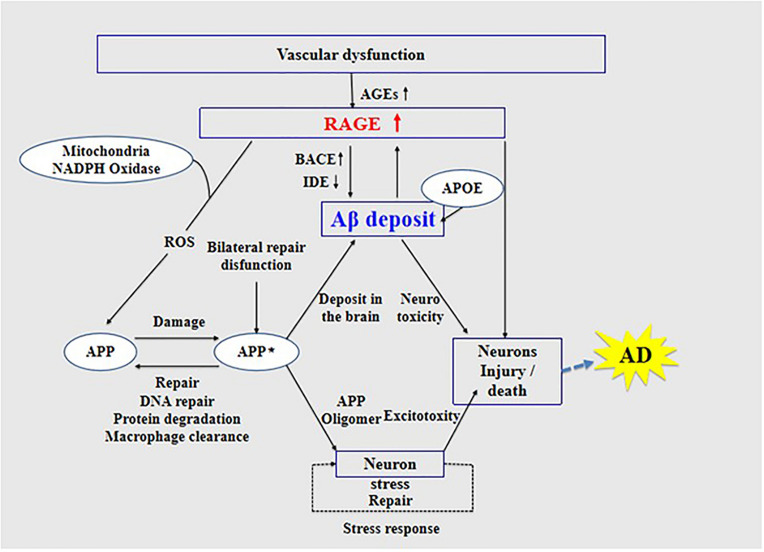
Receptor of advanced glycation end products (RAGE) mediates the possible mechanism of Aβ in the pathogenesis of Alzheimer’s disease.

High expression of RAGE is also harmful to the body. First, it activates the nuclear factor κB, further increasing the expression of RAGE and forming a positive feedback effect on inflammation (Wan et al., 2015; Fang et al., 2018). Second, it increases the expression of nuclear factor-1 in activated T-cells and of amyloid precursor protein (APP) β-site cleavage enzyme 1 (also known as BACE1), an important enzyme that cleaves amyloid precursors (Yan et al., 1996; Fang et al., 2010; Guglielmotto et al., 2012; Galasko et al., 2014). Increased BACE1 activity increases Aβ production (Maesako et al., 2019). In addition, Aβ can activate RAGE, increasing the expression of pro-inflammatory cytokines like TNF-α, interleukin 6 (IL-6), and macrophage colony-stimulating factor (Dukic-Stefanovic et al., 2003). In turn, RAGE activation exacerbates the production and aggregation of Aβ and the formation of NFTs and destroys synaptic transmission and neurons (Cai et al., 2016) (see Figure 3), which promote the occurrence and development of AD.

Inhibition of RAGE can prevent Aβ damage in nerve cells and cerebral vasculature. The possible mechanism of RAGE function in AD provides a theoretical basis and new ideas for the early diagnosis of AD and development of new drugs for the prevention and treatment of AD.

### RAGE and RAGE-Targeting Brain Imaging

Many studies have shown that AGEs are important in neurodegenerative diseases (Li J. et al., 2012; Nenna et al., 2015), while *in vitro* and *in vivo* studies have demonstrated the potential of RAGE as a receptor for AGE and as a therapeutic target in neurodegeneration (Sparvero et al., 2009; Deane et al., 2012; Nasser et al., 2015; Ray et al., 2016). Receptor for advanced glycation end products PET imaging has also been proven to assist in the diagnosis and treatment of neurodegenerative diseases (Kim et al., 2018; Konopka et al., 2018; Goldklang et al., 2019). The full-length human RAGE consists of three domains, namely, the extracellular, the hydrophobic transmembrane, and the cytoplasmic domains, while the main binding domain structure V is located on the extracellular part of the receptor (Bongarzone et al., 2017). Receptor for advanced glycation end products is expressed in a regulated manner, at low levels, in most differentiated adult cells, whereas its expression is high in embryonic cells (Demling et al., 2006). Moreover, RAGE is highly expressed in many inflammation-related pathological states such as vascular disease, diabetes, and neurodegeneration (Hudson et al., 2008; Sparvero et al., 2009). It is important in Aβ-mediated neurotoxicity (Piras et al., 2014), and its signaling pathway is also essential in AGE-induced tau phosphorylation and spatial memory impairment (Choi et al., 2014). Studies using murine models of chronic disease have demonstrated the involvement of RAGE in pathophysiological processes by means of a receptor decoy of soluble RAGE (Bierhaus et al., 2005). Moreover, RAGE was found to be relatively increased on the membrane of neurons and microglia in AD-related neuronal dysfunction (Yan et al., 1996; Cai et al., 2016). Considering the key functions of RAGE, there is a need for molecular imaging agents to measure RAGE expression in neurodegenerative diseases.

For developing novel RAGE inhibitors as potential AD therapeutics, Han et al. (2014) designed and synthesized a series of pyrazole-5-carboxamides to screen for excellent RAGE inhibitors. Screening identified a 4-fluorophenoxy analog with significant brain Aβ-lowering effects, as well as favorable aqueous solubility named 40, which were determined to be excellent RAGE inhibitors. Deane et al. (2012) synthesized a high-affinity RAGE-specific inhibitor, FPS-ZM1, which was selected after screening a second-generation library of compounds designed based on the common structural features of three leading compounds in a primary screen. Compared to other analogs (e.g., FPS1, FPS2, and FPS3), the functional groups of the leading tertiary amides in FPS-ZM1 were altered to reduce the molecular weight to less than 450 Da and decrease the number of hydrogen bonds. FPS-ZM1 has a molecular weight of 327 Da and 1 H-bond (see Table 1). The authors verified its effect using APP^sw/0^ mice, an AD model, and found that it can cross the BBB, acts on the V-type region of RAGE, and can still bind to RAGE after crossing the BBB, thereby blocking the role of intracranial RAGE (Lv et al., 2015; Hong et al., 2016). These results indicated the guaranteed binding ability of FPS-ZM1. In addition, FPS-ZM1 was shown to completely restore cerebral blood flow, inhibit neurotoxicity, microglial activity, and the neuroinflammatory response and improve cognitive behavior. Moreover, FPS-ZM1 has a wide safety range, with no toxic effects, even when using doses 500 times higher than the therapeutic dose (Deane et al., 2012). The above suggest that FPS-ZM1 is a potent multimodal RAGE blocker that effectively controls the progression of Aβ-mediated neurodegeneration and, thus, may be used as a disease-modifying agent for AD.

**TABLE 1:** Features of FPS1-3 and FPS-ZM1.

	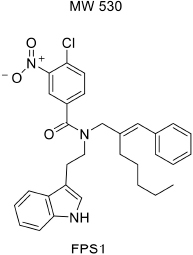	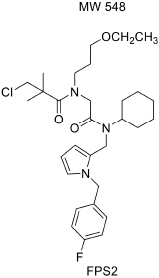	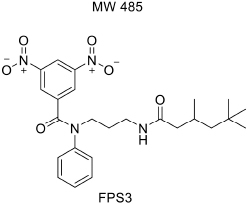	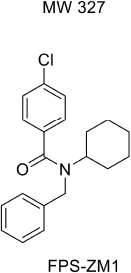

MW	530	548	458	327
K*i* (nM)	208 ± 12^A^	146 ± 21^A^	50 ± 9	25 ± 9^A^
K*i*/K*d*	2.78 ± 0.17^A^	1.94 ± 0.21^A^	0.66 ± 0.09	0.34 ± 0.04^A^
PS product (μl/g/min)	ND	ND	0.35 ± 0.10	18.67 ± 2.78
Brain uptake (%)	ND	ND	0.71 ± 0.20	37.34 ± 5.56

*MW, molecular weight; Ki, inhibitory constant; Kd, dissociation constant; PS, permeability surface area.*

Based on the importance of the RAGE signaling pathway in AGE-induced tau phosphorylation and spatial memory impairment, research and development of imaging agents with characteristics that can reflect the early pathological mechanism of AD is a highly active field. According to the above characteristics of FPS-ZM1, Cary et al. (2016) synthesized the first small-molecule BBB-permeable PET radioligand for RAGE, [^18^F] RAGER, and conducted a preliminary preclinical study. Micro-PET imaging in rodents and non-human primates indicated that [^18^F] RAGER clusters in the expression area of RAGE, while further molecular docking experiments determined the binding site of RAGER, indicating that [^18^F] RAGER and RAGE distribution area colocalization may have a binding effect. Kong et al. (2016) identified a new [^18^F]-FPS-ZM1 probe targeting RAGE among thousands of small molecules by testing different radiolabeling methods. The probe was radioactively synthesized with a purity of up to 99% and an activity of 30 mCi/ml and was shown to be lipophilic. The authors also studied the probe’s hemodynamics and verified its safety by performing animal experiments. They found that the low-molecular-weight [^18^F]-FPS-ZM1 is stable, electrically neutral, lipophilic, and weight independent. Micro-PET imaging and autoradiography results also indicated that [^18^F]-FPS-ZM1 is a promising RAGE-specific probe.

Several studies have documented other PET imaging probes for early AD diagnosis, such as those aimed at various targets, including Aβ, tau, and others. Tau-targeting imaging probes such as [^18^F]-THK-5351, [^18^F]-THK-5117, and [^18^F]-AV-1451 show a high uptake in the patients’ cortex (Harada et al., 2015; Lemoine et al., 2015; Passamonti et al., 2017; Kobayashi et al., 2018; Valotassiou et al., 2018), which means that they can accurately detect NFTs, thus helping in the early diagnosis of AD. At the same time, [^11^C] PiB, an analog of thioflavin-T and a benzothiazole derivative, was the first probe specifically targeting Aβ (Rabinovici et al., 2007; Lim et al., 2014; Lemoine et al., 2015; Kobayashi et al., 2018). Since then, many new probes targeting Aβ have appeared, including [^18^F]-florbetapir, [^18^F]-florbetaben, and [^18^F]-flutemetamol, all showing high affinity and specificity for Aβ (Valotassiou et al., 2018). These tau and Aβ-targeting probes can also be used for quantification analysis to further validate the role of RAGE in the pathogenesis of AD (Fang et al., 2018). As RAGE overexpression precedes Aβ plaque formation (Luzi et al., 2020), [^18^F]-FPS-ZM1 PET/CT imaging is expected to be more sensitive than traditional Aβ imaging. It can monitor changes in cerebrovascular function over time and thus provide accurate, reliable, and reproducible non-invasive *in vivo* quantitative data for local or whole-brain pathological changes.

Although many tracers have been developed to aid in the diagnosis and treatment of AD, including those targeting tau, P2X7, phosphodiesterase PDE10A, and synaptic vesicle glycoprotein 2A (McCluskey et al., 2020), only few RAGE-targeting imaging tracers are currently available apart from [^18^F]-FPS-ZM1, and they all have certain limitations in the diagnosis of AD. Available RAGE probes include the ^99m^Tc-F(ab′)_2_ anti-RAGE fragment developed by Tekabe et al. (Shan, 2004; Tekabe et al., 2010), which has only been applied in atherosclerosis and peripheral arterial disease, but not in AD. Another probe was developed by Hoppmann et al. (2008) on the basis of a multigenic family of Ca^2+^-modulated proteins, namely, S100, as RAGE ligands. However, compared with the high affinity and specificity of FPS-ZM1 for RAGE, this probe lacks stability and has low affinity for RAGE (Wolf et al., 2011). Recently, another RAGE-targeting probe, ^64^Cu-Rho-G4-CML, was developed by Konopka et al. (2018), which may be the best RAGE-targeting imaging agent currently available for cancer. However, its size prevents it from crossing the BBB, rendering it ineffective for neurological assessments (Konopka et al., 2018). Compared with these three probes, [^18^F]-F PS-ZM1 is expected to be more potent and could greatly improve early diagnosis, prevention, screening, and evaluation of AD and could help develop an imaging agent with appropriate characteristics that can reflect the early pathological mechanism of AD (Table 2).

**TABLE 2:** PET and SPECT radioligands for imaging RAGE.

Radiotracer	Method	Leading compound	Applied disease	References
^99m^Tc-F(ab′)_2_	SPECT	Polyclonal antibody to RAGE	Atherosclerosis and peripheral arterial disease	Shan, 2004; Tekabe et al., 2010
^18^F-S100	PET	A multigenic family of Ca^2+^-modulated proteins (S100)	No related reports	Hoppmann et al., 2008; Wolf et al., 2011
^64^Cu-Rho-G4-CML	PET	Carboxymethyl-lysine-modified human serum albumin	Cancer	Konopka et al., 2018
^18^F-FPS-ZM1/18FRAGER	PET	RAGE-specific inhibitor (FPS-ZM1)	Alzheimer’s disease	Lv et al., 2015; Kong et al., 2016

*RAGE, receptor of advanced glycation end products; SPECT, single-photon emission computed tomography; PET, positron emission tomography.*

## Conclusion

There is currently no breakthrough drug treatment for AD, which has become a serious social and economic problem. Although the progression of AD cannot be prevented or reversed, the availability of radioactive tracers for RAGE PET imaging will allow us to monitor RAGE brain expression levels in AD. Receptor for advanced glycation end products has an important role in the development of AD, but the kind of state RAGE exists in AD and the way it acts on Aβ and tau have yet to be determined. It is unclear whether increased RAGE expression affects the behavior and pathophysiology of AD models. Thus, an in-depth study of the mechanism of action of RAGE is essential for the further understanding of neurological diseases.

In this review, we introduced the RAGE-targeting probe [^18^F]-FPS-ZM1. Compared with probes targeting Aβ and the tau protein, [^18^F]-FPS-ZM1 has advantages in exploring the involvement of RAGE in AD pathogenesis. Due to its high specificity and affinity for RAGE, [^18^F]-FPS-ZM1 is believed to provide accurate and reliable *in vivo* data for studying local or whole-brain pathological changes. Thus, [^18^F]-FPS-ZM1 could greatly promote the early diagnosis and evaluation of AD and provide a way to reflect the early pathological mechanism of AD.

## Author Contributions

YK, BS, JW, and YG guided the study. YK, CL, YZ, JQ, and CZ wrote the manuscript. All authors contributed to the article and approved the submitted version.

## Conflict of Interest

The authors declare that the research was conducted in the absence of any commercial or financial relationships that could be construed as a potential conflict of interest.
